# Assessing the Likelihood of High Pathogenicity Avian Influenza Incursion Into the Gamebird Sector in Great Britain *via* Designated Hatcheries

**DOI:** 10.3389/fvets.2022.877197

**Published:** 2022-04-22

**Authors:** Mayumi Fujiwara, Harriet Auty, Ian Brown, Lisa Boden

**Affiliations:** ^1^Global Academy of Agriculture and Food Security, Royal (Dick) School of Veterinary Studies, University of Edinburgh, Edinburgh, United Kingdom; ^2^Institute of Biodiversity, College of Medical, Veterinary & Life Sciences, Animal Health and Comparative Medicine, University of Glasgow, Glasgow, United Kingdom; ^3^Animal and Plant Health Agency, Weybridge, United Kingdom; ^4^School of Veterinary Medicine and Science, University of Nottingham, Nottingham, United Kingdom; ^5^Royal Veterinary College, University of London, London, United Kingdom

**Keywords:** Avian Influenza, risk assessment, HPAI, gamebird, hatchery

## Abstract

The outbreaks of High Pathogenicity Avian Influenza (HPAI) in the United Kingdom in 2017 and 2021 had a substantial impact on the gamebird industry and highlighted to policymakers the importance of existing knowledge gaps for effective disease control. Despite the size of the industry, the impact of HPAI on the gamebird industry is not well-understood. To improve future disease preparedness, a veterinary risk assessment to explore the risk of HPAI incursion into the gamebird sector in Great Britain *via* a designated hatchery was commissioned by Scottish Government Animal Health and Welfare Division. Hatchery designation is a legal requirement for hatcheries located within disease control zones or that have business links to premises located in disease control zones to continue operating during an HPAI outbreak. Several risk pathways were identified, which involved various management procedures associated with egg production through to the delivery of day-old chicks. The overall likelihood of the HPAI virus introduction into a designated hatchery through hatching egg movement is considered to be low (high uncertainty). The overall likelihood of onward transmission of the HPAI virus into gamebird rearing sites from a designated hatchery through day-old chick movement is also considered to be low (medium uncertainty). These risk levels are based on the assumption that relevant control measures are observed, as enhanced biosecurity is one of the requirements for hatchery designation. However, high uncertainties and variabilities were identified in the level of compliance with these biosecurity measures. Factors increasing the likelihood level include management practices typical to this sector, such as having multiple egg production sites, raising birds at outdoor sites, catching birds from the wild for egg production, having various scale of satellite farms in various locations, importing eggs and day-old chicks from overseas, as well as the proximity of the game farm to the infected premise or to higher risk areas. This study offers evidence for policymakers to help develop criteria for hatchery designation and proposes important mitigation strategies for future disease outbreaks specific for the gamebird sector.

## Introduction

Avian Influenza (AI), also known as bird flu, is a notifiable poultry disease in the United Kingdom (UK) (1) which causes infectious viral disease in birds including domestic poultry. AI viruses (AIVs) can be classified as low pathogenic and high pathogenic, based on the pathogenicity in chickens, and poultry species are susceptible to most AIV strains. High pathogenicity AI (HPAI) virus normally causes high mortality and severe clinical signs, whilst an infection with low pathogenicity AI (LPAI) virus causes asymptomatic or mild respiratory disease (2). Wild waterfowl are usually considered as a natural reservoir of both low and high pathogenicity AI, and they can transmit the virus to domestic poultry *via* direct and indirect contact.

Since September 2021, an unprecedentedly high number of HPAIV detections have been reported in poultry, captive and wild birds in Europe, including 83 HPAI H5N1 infections in commercial and captive poultry premises in the UK (as of 31st January 2022). This resulted in a Great Britain (GB)-wide Avian Influenza Prevention Zone (AIPZ) introduction on 29th November 2021. HPAI outbreaks in the UK in the 2020/21 winter also affected 20 domestic poultry premises and four non-poultry captive bird premises by infection with the H5N8 or H5N1 virus strains, including two gamebird rearing premises in Scotland and Wales (3). In other European countries, a number of HPAI outbreaks in domestic poultry have reported in 2020/21 and 2021/22 winter, including gamebird holdings in Denmark and Finland in 2020/21 (4) and Denmark (5) and Czech Republic (6) in 2021/22. HPAI outbreaks in the UK in 2016/17 also affected three gamebird rearing premises, belonging to a large gamebird hatchery (7). The outbreak was initially confirmed in the gamebird rearing site but spread to other breeding sites within the same operator *via* movement of infected birds (8). This resulted in the culling of approximately 80,000 mixed gamebird parent stock (pheasants, partridges, ducks and other birds) (7), and the farm was unable to produce eggs and supply day-old chicks to gamebird keepers, causing serious economic losses.

Gamebird shooting is a popular activity in the UK. Each year, at least 600,000 people shoot gamebirds and spend £2.5 billion on related goods and services (9). This spending contributes an annual gross value of £2 billion to the UK economy (10). Approximately 20% of gamebirds shot are wild, and the remaining 80% are reared on gamebird farms and released (10). It is estimated that approximately 35 million gamebirds are reared and released each year; the majority of these birds are pheasants (80%), followed by red-legged partridges (16–17%) (11). There are over 20,000 gamebird premises and around 300 recognized gamebird farms across the UK (10, 12). Most common species reared on those farms are pheasants and partridges, and some farms keep more than 25,000 birds.

The AI outbreak in the gamebird sector in 2016/17 highlighted to policymakers important knowledge gaps in the gamebird industry and gamebird behavior (7). Despite the size of the industry, the impact of HPAI incursion into the UK gamebird sector is not well-understood and has only been investigated following clinical disease suspicion in affected birds. A single gamebird operator can have multiple production sites at different locations across the country, and movements of birds, eggs, people, and vehicle/equipment between these premises are likely to occur during the gamebird production period. Gamebird hatcheries, for example, receive hatching eggs from and send day-old chicks to multiple premises including operators within their own business and business partners. This involves frequent movements of live birds, eggs, people, and vehicle/equipment between the premises. Therefore, gamebird hatcheries can be a potential source of disease spread, which can lead to impacts on all the operations linked to this premise.

During an outbreak of AI, various forms of disease controls are put in place (1, 13). If an AI outbreak caused by H5 or H7 virus subtypes is confirmed in a domestic bird premise (any bird species), disease control zones are imposed around the infected premises (IP), and movements of birds and bird products (e.g., eggs) are prohibited within these zones without specific licenses. There is a legal requirement that a premise located within the zones or a premise with business links to a site within the zones is officially designated by the local government authority in order to continue normal business operation during an outbreak (1, 13). Therefore, gamebird hatcheries that are within disease control zones or receive eggs/send day-old chicks to premises located within the zones cannot operate without designation.

The hatchery designation process includes official inspection and approval, with enhanced biosecurity and additional requirements (e.g., special marking, record keeping) being necessary for approval. The current designation criteria were developed based on commercial poultry farms, which may not be suitable for the gamebird sector due to differences in their management practices and business styles compared to the commercial poultry sector.

In this study, veterinary risk assessments (VRA) were developed to evaluate the likelihood of HPAI incursion into the gamebird sector in GB during an HPAI outbreak *via* a designated hatchery. These VRAs aimed to underpin the process of gamebird hatchery designation. Several risk pathways were identified, that were associated with the movement of hatching eggs, day-old chicks, people (staff/visitors), and vehicle/equipment between designated hatcheries and other production sites during an outbreak. This paper describes the risk assessments and conclusions, as well as highlighting key assumptions and knowledge gaps.

## Materials and Methods

Two risk questions were developed; (1) in an outbreak of HPAI, what is the likelihood of HPAI introduction into a designated hatchery in GB through movements of hatching eggs from gamebird egg production sites, and (2) what is the likelihood of HPAI introduction into gamebird rearing sites in GB through movements of day-old chicks from the designated hatcheries?

The first question consisted of two sub-questions; (1.1) what is the likelihood of HPAI introduction into a designated hatchery through movements of hatching eggs from gamebird egg production sites located in the protection zone (PZ) and surveillance zone (SZ), or restricted zone (RZ: in the case of H5N1 outbreak in poultry in Scotland) and (1.2) what is the likelihood of HPAI introduction into a designated hatchery located in the PZ, SZ, or RZ through movements of hatching eggs from gamebird egg production sites located in disease-free zone?

We took a qualitative approach based on the framework developed by the World Organization for Animal Health (OIE) (14, 15), which included (i) hazard identification, (ii) risk pathway, (iii) legislation, definitions and assumptions, (iv) entry and exposure assessment, (v) consequence assessment, and (vi) overall likelihood levels (16). We considered the impact of different existing risk management options on these likelihoods. A qualitative approach was chosen over a quantitative risk assessment approach because of the lack of published data to quantitatively assess the probability of each event occurring [e.g., (16–18)]. Definitions of qualitative likelihood levels used were taken from the OIE framework (14), which were also adopted by the UK Department for Environment, Food, and Rural Affairs (DEFRA) (19) (Table 1).

**Table 1:** Definition of qualitative likelihood estimate levels.

**Likelihood level**	**Description**
Negligible	So rare that it does not merit consideration
Very low	Very rare but cannot be excluded
Low	Rare but could occur
Medium	Occurs regularly
High	Occurs very often
Very high	Events occur almost certainly

In order to develop risk pathways, it was essential to understand the gamebird industry structure. We visited the website of major gamebird companies, gamebird associations and DEFRA to obtain basic information about the industry and to access to industry and government publications (9–12, 20, 21). The gamebird hatchery visit was arranged to develop our understanding of typical gamebird management practices and to ground-truth the information obtained from the website. A follow-up questionnaire was sent to the farm manager to obtain more detailed information about industry practices (Supplementary Material). The drafted gamebird industry structure was reviewed by the industry representative from the Game Farmers' Association (GFA) and was modified based on the feedback received.

Risk pathways were developed, which outline the pathways leading to the introduction of and exposure to HPAIV. We reviewed the available literature including peer-reviewed publications, primary and secondary sources of data, and non-peer-reviewed publications to identify all relevant factors, which were likely to influence likelihood levels for each pathway. The literature search through the available databases was conducted using the following search terms: “Avian Influenza” AND “gamebird OR game bird,” “Avian Influenza” AND “pheasant OR pheasants OR partridge OR partridges OR duck OR ducks,” “Avian Influenza” AND “Galliforms OR Anseriformes.” The search results included peer-reviewed publications on both gamebird species and non-gamebird species (e.g., HPAI in chicken and turkey) where relevant, as well as non-peer-reviewed publications such as government reports on past/on-going outbreaks of Avian Influenza in the UK and Europe, national epidemiology reports, risk assessments and disease control strategies. Important references were also identified from review papers (2, 22). We reviewed the relevant and available literature to identify all relevant factors, which were likely to influence likelihood levels for each pathway.

Key knowledge gaps and/or areas of uncertainty were identified for each step during this process. Likelihood estimates for each step were developed based on the information available. We assumed that there is full compliance with preventive biosecurity measures for AI (23–25). However, there may be a large variation in the level of compliance between operators in reality, which might affect the risk level. Therefore, areas of particular concern for non-compliance were highlighted in the risk assessment. The consequences of a virus introduction into the gamebird hatchery and subsequent transmission to the rearing site as a result of the risk pathway were considered, and overall likelihood were determined based on a combination of the likelihood of exposure and release and the level of uncertainties (14). The VRAs were reviewed by the Scottish Government, the GFA, DEFRA and Animal Plant Health Agency, which led to adding more detailed (and unpublished) information about epidemiological findings from the past HPAI outbreaks in GB.

## Results

### Structure of the Gamebird Sector in the UK

**Figure 1:**
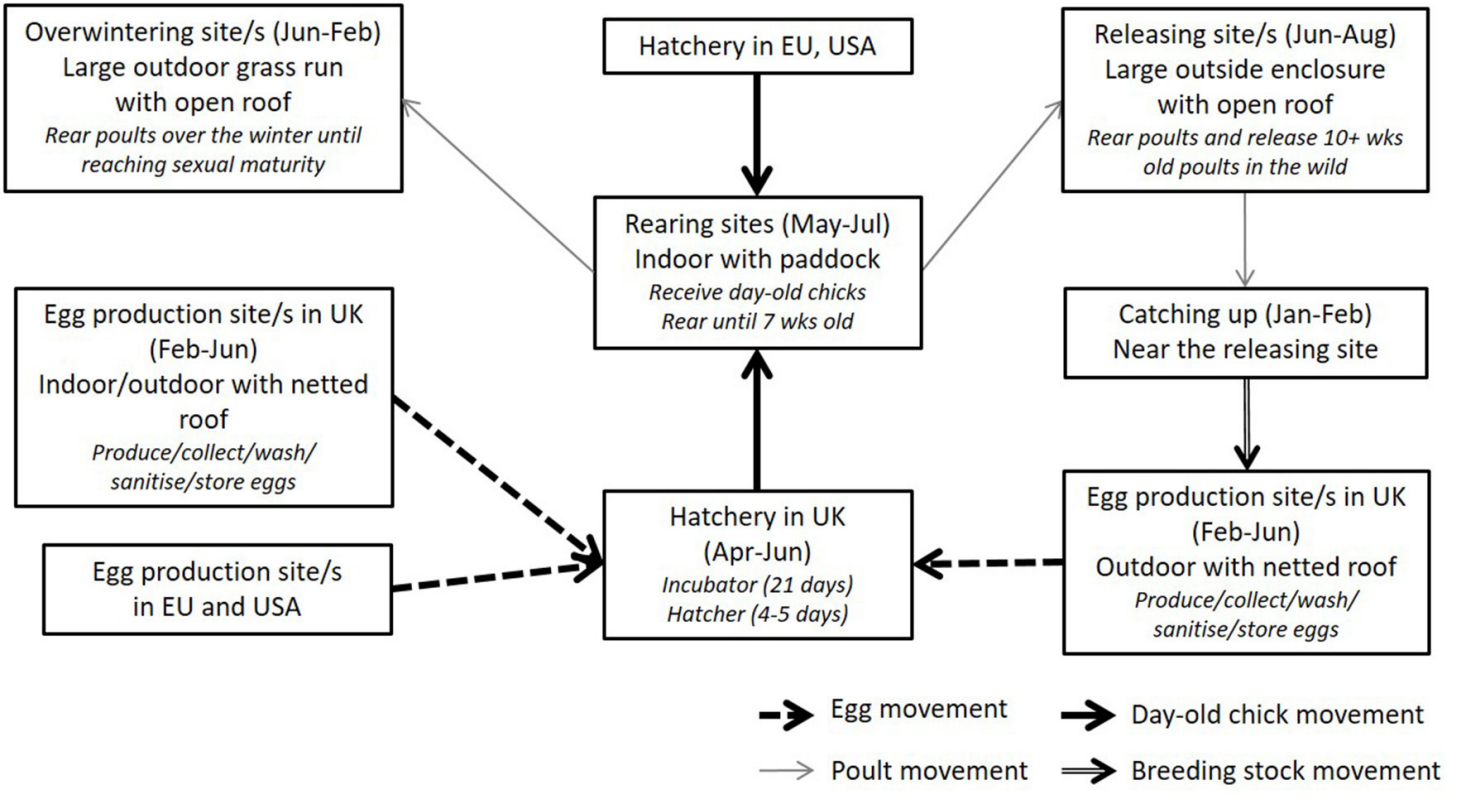
Summary of gamebird industry structure in the United Kingdom.

The most common gamebird species reared in GB are Galliformes species such as the Common pheasant (*Phasianus colchicus*) and the Red-legged partridge (*Alectoris rufa*). Only a few farms rear ducks. Geese and grouse species are not usually bred or reared in captivity for release, although wild birds are shot. Gamebirds normally start laying eggs from early March until mid-June. This may vary depending on the weather in each year, locations (e.g., England is normally earlier than Scotland), and species (e.g., partridges are 2 weeks behind pheasants). Eggs laid in the first 1–2 weeks may be discarded as they are normally infertile or low quality. Eggs are normally laid on the ground, which can be dirty, unless a raised laying unit is used (*pers comm* with the industry representative). Eggs are collected every day, washed, stored and delivered to a hatchery (on-site or at a different location) once per week. First fertile eggs normally start hatching from end-April to early May. Day-old chicks are transferred to a rearing site and reared until 7 weeks old (May–July). The birds are then moved to a releasing pen, which is a large outdoor enclosure with no roof (from June). At 10–11 weeks old (June–August), the birds can fly out in the wild but many released gamebirds stay close to the release pen (10). The overall industry structure is illustrated in Figure 1.

Gamekeepers purchase hatching eggs or day-old chicks from the gamebird farm and rear them on their site until releasing. Some gamekeepers produce eggs from their own flock by keeping poults over-wintered and/or catching adult birds from the wild that have survived until the end of the shooting season (pheasants only) for breeding. The breeding flock is kept at the egg production sites (indoor or outdoor with netted roof, or housed in laying cages, with a cock-hen ratio of 1:10 or more: Figure 1) from January or February until the end of breeding season (June). Capturing of survived gamebirds becomes illegal after 1 February in England (EN), Wales (W), and Northern Ireland (NI) and after 28 February in Scotland (S) under the Game Act of 1831 and the Game Preservation Act (Northern Ireland) 1928 (21).

Gamekeepers who do not have brooding accommodation or hatchery equipment purchase poults at 6–8 weeks of age or even older and place them in release pens. Gamekeepers breed and rear ~50% of birds in the UK, and the remaining 50% of birds reared come from recognized game farms. There are around 300 recognized game farms in the UK, which focus on selling day-old chicks and/or 6–8 week old poults (occasionally hatching eggs) to gamekeepers. They do not release birds but keep breeding stock over-wintered for egg production (June–February). Game farms who do not keep breeding stock may source eggs from other game farms (including game farms in Europe).

Around 40% of gamebirds released in the UK originate from EU member states, being imported as fertile eggs or day-old chicks (10, 12). There is a small trade of 6–8 weeks old poults from France, Scandinavian countries and the USA, although this accounts for 1–2% of released gamebirds population. Because of this structure, one hatchery may accept eggs from their own satellite farms and/or purchase eggs from other producers from inside and/or outside UK (e.g., France, Spain). One operator can have all the production stages at the same site or can have multiple sites at varying production stages [see (8, 10)]. Laying, rearing, and releasing sites may be also separated from each other.

### Hazard Identification

The hazard is HPAI. To date, viruses shown to cause HPAI in susceptible species include H5 and H7 subtypes (26), although not all H5 and H7 viruses are virulent (27). This risk assessment does not specify AIV strains due to limited availability of literature on HPAI in gamebird species.

Galliformes are susceptible to most AIV strains (2, 28, 29), and mortality and clinical signs are variable depending on host species, virus strains, and other factors such as environmental conditions and infection routes (e.g., natural infection or experimental). For example, recent outbreaks of HPAI in the UK in 2021/22 (H5N1), 2020/21 (H5N1/H5N8), and 2016/17 (H5N8) affected chickens, turkeys, pheasants, and partridges showing sudden and marked mortality (3, 5, 6, 8). Reported clinical signs seen in pheasants and partridges included: lethargy, depression, nervous signs, torticollis, recumbency, ataxia, and attenuated motor functions (30). H7N1 infection caused increased mortality and signs of nervous disease in ducks, geese, guinea fowls, quails, pheasants, and partridges (31–33).

Previous outbreaks and experimental studies have shown that infection with HPAIV strains that cause high mortality for chicken or turkeys do not necessarily lead to high mortality and high morbidity in these gamebird species (34–36). Contradictory results have also been reported between experimental inoculation and natural infection. The experimental inoculation of H5N2 (HPAI) that caused high mortality to chickens only caused mild and transient illness in pheasants (35) whereas natural infection with H5N2 in upland game farms in the United States caused 10% mortality and clinical symptoms in ring-necked pheasants (37). Different degrees of mortality or morbidity were observed in ducks between 2 and 5 weeks old after experimental infection with four different strains of H5N1 virus (38), whilst no clinical signs of disease or mortality were observed in H5N1 inoculated ducks (39). There was a significant decrease in egg production in the first week after inoculation but the quality of eggs, fertility rates, and duckling growth rates were not affected by the infection (39).

Birds with asymptomatic infection with AIV shed virus in their feces for up 10–15 days post-infection [e.g., HPAI H5N2: (35)] or longer (28, 34). It has been reported that bird-to-bird transmissions or indirect contact *via* contaminated water extended a viral shedding period for up to 45 days post-infection (28). Galliforme gamebirds (pheasants and partridges) normally show poor transmissibility, but ducks and geese can transmit viruses efficiently without clinical signs (*pers comm* with the industry expert). The virus can survive in the environment for a long period of time. For example, H5N2 virus was isolated from dead pheasants stored at 4°C for 23 days (35). H5N8 virus isolated from 2020/21 outbreaks retained infectivity in the environment up to 21 days at 4°C and 8.4 days at 20^o^C, which is longer than the H5N8 isolates from 2016/17 outbreaks (40).

### Risk Pathways

**Figure 2:**
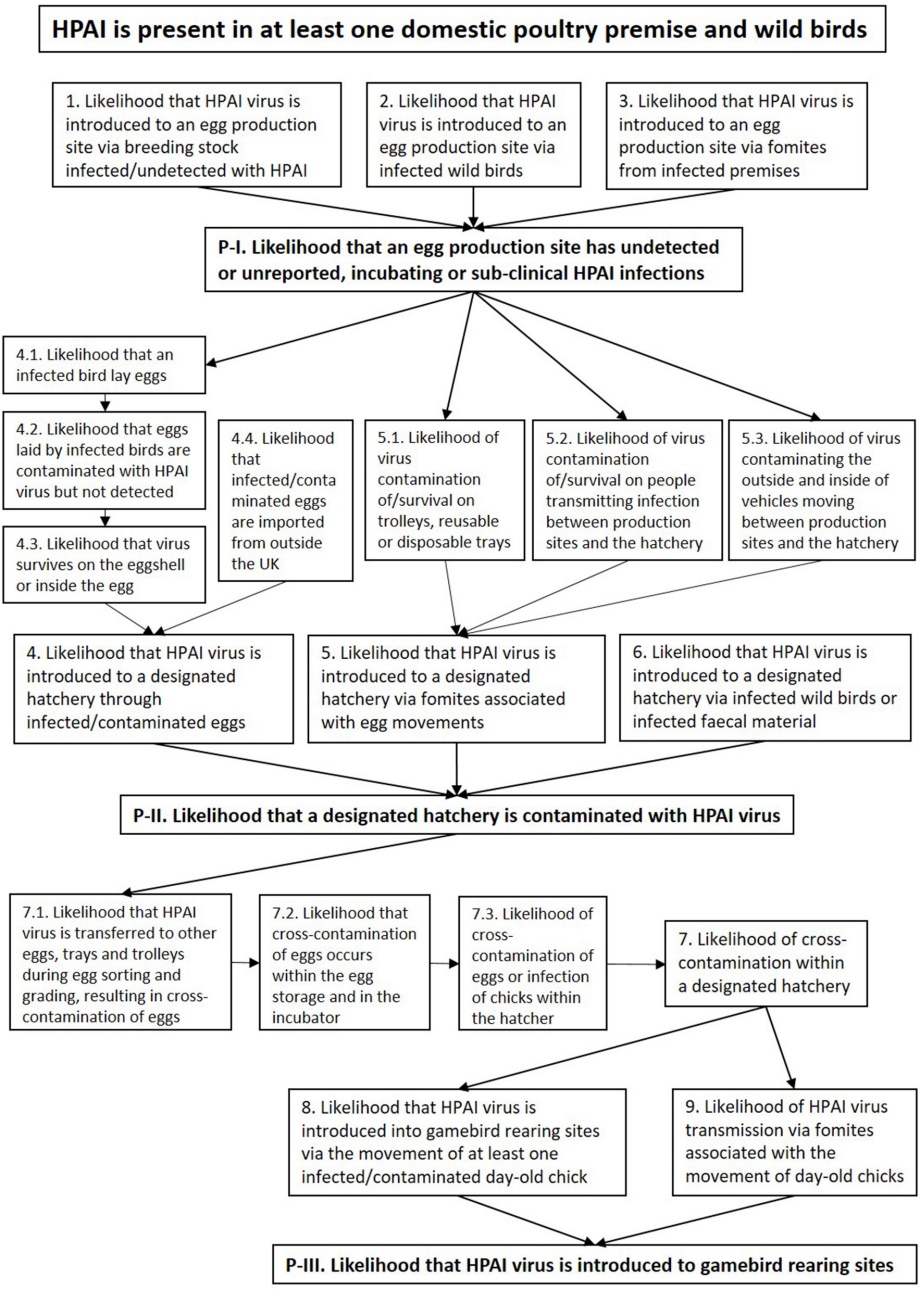
Summary of risk pathways, illustrating the steps of the exposure of HPAIV to susceptible birds and products at different gamebird production sites. P-I, P-II, and P-III indicate three pathways that are identified as enabling the virus to be introduced to a designated hatchery and ultimately spreading the virus to gamebird rearing sites through the movement of hatching eggs and day-old chicks. Number 1–9 indicate sub-pathways, with the numbers 4, 5, and 7 including further paths contributing to each of the sub-pathways.

Risk pathways were developed for each step of the exposure assessments. A summary risk pathway is shown in Figure 2. In each of the pathways we assume that an incursion of HPAI has already occurred in the country (i.e., entry has already occurred) and is present in at least one domestic poultry premise and wild birds, which may be at a varying stage of diagnosis, slaughter, cleansing, and disinfection (C&D).

### Legislation and Definitions

**Figure 3:**
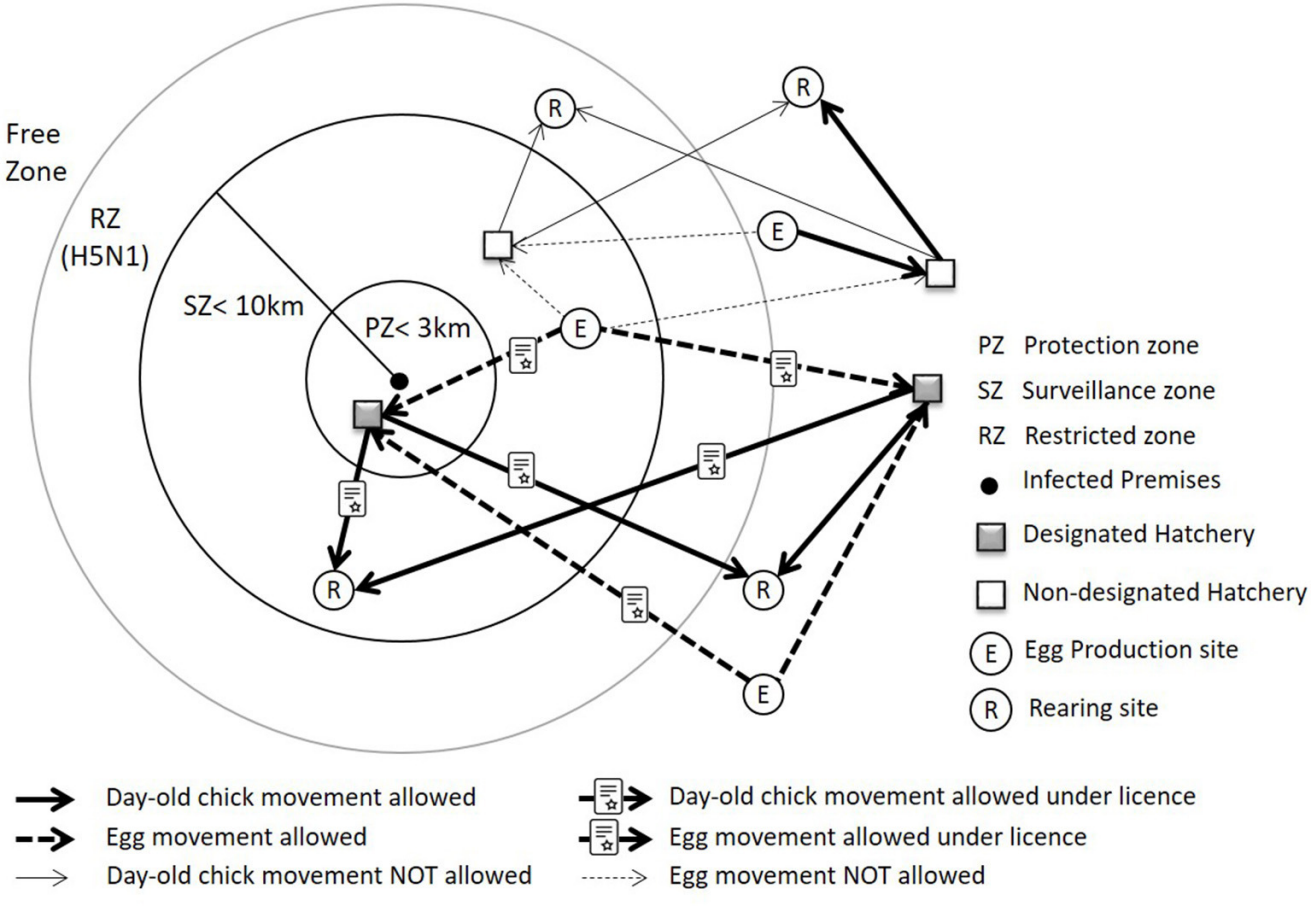
Disease control zones during an outbreak, and illustration of allowed and not-allowed movement of eggs and day-old chicks within and between the zones.

Disease control zones (Figure 3): Statutory disease control requirements apply to premises on suspicion and confirmation of AI. On confirmation of disease, a Protection Zone (PZ) of minimum radius 3 kilometers (km) and Surveillance Zone (SZ) of minimum radius 10 km are implemented which place restrictions on movements and activities around IP to prevent the spread of disease (1, 13). In the case of H5N1 HPAI in poultry in Scotland, a Restriction Zone (RZ) may be declared, but the size is not prescribed (1). In the case of H5N1 AI in wild birds in Scotland, wild bird control areas (WBCA) and wild bird monitoring areas (WBMA) may be declared, and the minimum size of the control areas are determined during the course of an outbreak.

Hatchery designation: During an outbreak of HPAI, there is a requirement for hatcheries to be designated if they are in a PZ/SZ/RZ/WBCA/WBMA and intend to accept eggs and/or send day-old chicks from/to other premises. A hatchery must also be designated if it is located in the free area but receives hatching eggs from egg production site(s) in the PZ/SZ/RZ/WBCA/WBMA. Day-old chicks can be sent to premises located in the PZ/SZ/RZ/WBCA/WBMA only when they originate from a designated hatchery. The criteria for hatchery designation include enhanced biosecurity and additional requirements such as adequate record-keeping/traceability, separation of restricted production, and special marking of production (1, 13).

There is also a requirement for licenses if poultry and eggs are to be moved within/between the zones. Licensed moves may be under general or specific licenses depending on the location of the source farm, the location of the hatchery and current outbreak conditions. The term “poultry” in EU Council Directive 2005/94/EC includes “all birds that are reared or kept in captivity for the production for meat or eggs for consumption, the production of other products, for restocking supplies of gamebirds or for the purposes of any breeding programme for the production of these categories of birds.”

Gamebird farms may have all of their sites with different production stages at the same premises, or they have multiple locations with different production stages. If eggs and day-old chicks do not leave their premises (all the sites in the same location), there is no requirement for a hatchery to be designated, even though it is located within the PZ/SZ/RZ/WBCA/WBMA. However, the current legislation does not specify the exact distance between premises where the hatchery designation is required.

### Assumptions

(1) An HPAI outbreak has been detected and confirmed in domestic birds in GB. AI is assumed to be present in the wild bird population (possibly undetected).
(2) Egg production sites (i.e., breeding operations where laying stock is kept to produce eggs) within or outside of control zones may have undetected HPAI in their flock(s). If HPAI has been detected, movement of eggs and material from the source farm would not be permitted and the farm would be depopulated. If the source farm was proven to be free from disease with absolute certainty (i.e., the results of epidemiological enquiries were confident that tracings were complete and there had been no onward spread), there would be no risk.
(3) Before the confirmation of and at the beginning of the outbreak, some production sites may hold infected but undetected breeding stock (parent birds) that had been captured in the HRA or PZ, and eggs produced in this site may have been delivered to both designated and non-designated hatcheries.
(4) A hatchery needs to be designated when a confirmed outbreak has occurred in at least one source farm of hatching eggs or one destination farm for that hatchery (where day-old chicks are sent) and/or the hatchery itself is within a disease control zone (i.e., the PZ, SZ, or RZ) or a WBCA/WBMA.
(5) In an outbreak, current legislation dictates that movement of eggs and day-old chicks can only occur directly between a premise and a hatchery.
(6) If the hatchery is designated because an egg production site is located within a zone, this does not preclude other, multiple production sites in free areas (i.e., not in a zone) from also sending their eggs to the designated hatchery.
(7) There is full compliance with Government-recommended preventive biosecurity measures for Avian Influenza (AI) (23–25).
(8) This risk assessment focuses on the risk of transferring infection between a production site and a hatchery, within a hatchery, and between a hatchery and a rearing premise. Onward risks of transmission to other susceptible birds, humans or wildlife associated with the production or movement of hatching eggs and day-old chicks are outside the scope.
(9) The likelihood estimates reported for each step in the risk pathway represent the likelihood if there is full compliance with specified control measures.

### Exposure Assessment

Each step in the risk pathway is discussed below and key factors, uncertainties and likelihood levels for each step in the risk pathway are summarized in Table 2. Under the assumption that HPAI is present in at least one domestic poultry premise and wild birds, there are three pathways that enable the virus to be introduced to a designated hatchery and ultimately spread to gamebird rearing sites through the movement of hatching eggs and day-old chicks.

- Pathway I. The likelihood that an egg production site has undetected or unreported, incubating or sub-clinical HPAI infections.
- Pathway II. The likelihood that a designated hatchery is contaminated with HPAIV.
- Pathway III. The likelihood that HPAIV is introduced to gamebird rearing sites.

**Table 2:** Key factors, uncertainties, and likelihood levels for each step of the risk pathway.

**Factors affecting each step of risk pathway**	**Likelihood and uncertainty levels**	**Description**
**Pathway I. What is the likelihood that an egg production site has undetected or unreported, incubating or sub-clinical HPAI infections?**		
1. Likelihood that HPAIV is introduced to a production site *via* breeding stock infected/undetected with HPAI		
- Location of the habitat of released gamebirds and the over-wintering site - Presence of infected wild birds in the habitat of released gamebirds - Proximity of the habitat of released birds and the over-wintering site to infected premises (IP) - Housing type for over-wintering stock (e.g., outdoor access) - Length of infection, viral strains, latent period, incubation period, host species, clinical signs, mortality rates, and regular health inspection by farm staff - Amount and the duration of viral shedding after AI infection	Infection with mild/no clinical signs: **HIGH (high uncertainty)** Infection with high mortality and clinical signs: **VERY LOW (high uncertainty)**	At the beginning of an outbreak, infected/undetected breeding stock may have been caught up in a higher risk area (HRA) or near the IP It is uncertain whether transport vehicles, personnel and equipment shared with other operators and how rigorous the cleansing and disinfection (C&D) procedures are, whether infected birds show high mortality and clinical signs of disease, and how common it is to do regular health inspection at the entry of new birds into the egg production site Local transmission depends on host species, virus strain, and environment factors such as shared water source and weather condition
2. Likelihood that HPAIV is introduced to an egg production site *via* wild birds and their fecal materials		
- Location of the egg production site - Presence of infected wild birds - Housing system (e.g., outdoor/indoor)	Egg production site with outdoor access and (i) within 2 km from a HRA, (ii) within 2 km from PZ/WBCA, (iii) within an area where positive cases from wild bird surveillance: **MEDIUM (low uncertainty)** Egg production site with outdoor access but out of the zones above or indoor egg production site: **VERY LOW (low uncertainty)**	Wild birds may be the common source of AI infection in commercial gamebirds Likelihood may decrease toward the end of the winter migrating season (when the hatchery is the busiest)
3. Likelihood that HPAIV is introduced to an egg production site *via* fomites from IPs		
- Geographical and epidemiological proximity of egg production sites to the IP - Potential for cross-contamination *via* other premises	If the IP is not a commercial gamebird farm: **VERY LOW (medium uncertainty)** If the IP is a gamebird farm, - Different operator: **VERY LOW (medium uncertainty)** - Same operator: **MEDIUM** (medium uncertainty) - The risk will increase if they share staff, vehicles and equipment	Within the same operator, it is uncertain whether egg collection pathways and egg transport vehicle may be shared Biosecurity levels vary between farms
**Pathway II. What is the likelihood that a designated hatchery is contaminated with HPAIV?**		
4. Likelihood that HPAIV is introduced to a designated hatchery through infected/contaminated eggs		
4.1. Likelihood that an infected bird lays eggs		
- Virus strain, clinical symptoms	If the circulating AIV causes sub-clinical or no clinical signs: **HIGH (medium uncertainty)** If the circulating AIV causes high mortality, clinical signs and reduced egg production: **LOW (low uncertainty)**	Data on the egg production of infected gamebird species is scarce, but studies in the chicken suggest decrease in egg production is likely
4.2. Likelihood that eggs laid by infected birds are contaminated with HPAIV but not detected		
- Surface contamination depends on the amount of virus shed and its survival in the environment	Infection with high mortality and clinical signs: **LOW (high uncertainty)**—AI would be clinically detected before eggs departing the production site Infection with mild/no clinical signs: **MEDIUM (high uncertainty)**—Contaminated eggs can be delivered to the production site undetected (providing that the egg washing procedure is not effective)	Limited information on the possibility of egg contamination by AI infected gamebirds and the effectiveness of egg washing procedure in gamebird species
4.3. Likelihood that virus survives on the eggshell or inside the egg		
- Viability of HPAIV in the environment	**LOW (medium uncertainty)**	Uncertainty in the virus survival time within the egg. Egg washing could introduce contaminations into the egg
4.4. Likelihood that infected/contaminated eggs are imported from outside the UK		
- see above 4.1, 4.2 and 4.3 - Delayed detection of HPAI on source farms due to the absence of clinical sings or low mortality	No infection in the country of egg origin: **LOW (high uncertainty)** Infection in the country of egg origin: **MEDIUM (high uncertainty)**	Data lacking on how many eggs are purchased online from different countries
5. Likelihood that HPAI is introduced to a designated hatchery *via* fomites associated with egg movements from the production site		
5.1. Likelihood of virus contamination of/survival on trolleys, reusable or disposable trays		
- Type and material of egg trays used	**VERY LOW (medium uncertainty)**	No published data on the survival of virus on plastic egg trays, but adequate C&D should be sufficient to kill the virus on plastic or metal surfaces
5.2. Likelihood of virus contamination of/survival on people transmitting infection between production sites and the hatchery		
- Implementation of and compliance with biosecurity protocol, behavior and trainings	**LOW (medium uncertainty)**	Control measures are normally put in place, but the measures implemented vary between farms
5.3. Likelihood of virus contaminating the outside and inside of vehicles moving between production sites and the hatchery		
- Virus presence and survival in the environment - Frequency of vehicles deliveries between the side - Number of business links	**LOW (high uncertainty)**	The exact distance at which the hatchery is considered a separate location is not clearly defined. High uncertainty in the percentage of gamebird operators with multiple sites, whether egg collection pathways are shared, whether vehicles are dedicated for egg transportation only
6. Likelihood that HPAIV is introduced to a designated hatchery *via* infected wild birds or infected fecal material		
- Location of the hatchery - Presence of infected wild birds - Level of biosecurity measures	Hatchery located within 2 km from an HRA or within PZ/WBCA: **MEIDUM (medium uncertainty)** Within SZ/WBMA: **LOW (medium uncertainty)** Within RZ or disease free zones: **VERY LOW (medium uncertainty)**	Wild bird can be a most likely source of AI infection in gamebirds, but control measures implemented in the hatchery vary between operators
**Pathway III. What is the likelihood that HPAIV is introduced to gamebird rearing sites?**		
7. Likelihood of cross-contamination within a designated hatchery		
7.1. Likelihood that the HPAIV is transferred to other eggs, trays and trolleys during egg sorting/grading, resulting in cross-contamination of eggs		
- Viral load on the surface of the eggshell - Level of dirt on the egg - Method of egg transfer (mechanical or manual) - Proximity to neighboring eggs (touching or not) - Breakage of eggs	**LOW (high uncertainty)**	No published data on the survival of virus on plastic egg trays, and egg washing could introduce contaminants into the egg
7.2. Likelihood that cross-contamination of eggs occurs within the egg storage and in the incubator		
- Breakage of infected/contaminated eggs on a tray - Cross-contamination during candling	**LOW (low uncertainty)**	It is uncertain how often eggs break in the egg storage, but this could be avoidable with proper handling
7.3. Likelihood of cross-contamination of eggs or infection of chicks within the hatcher		
- Presence of at least one infected egg in the hatcher - Viral survival time on the egg surface	**LOW (medium uncertainty)**	Limited information on the hatchability of eggs from infected birds, and Post-hatching contamination/infection
8. Likelihood that HPAIV is introduced into gamebird rearing sites *via* the movement of at least one infected/contaminated day-old chick		
- Detection of infected chicks would be only possible with clinical signs and increased mortality	**LOW (medium uncertainty)**	Limited information on the likelihood of Post-hatching contamination/infection in chicks
9. Likelihood of HPAI transmission *via* fomites associated with the movement of day-old chicks		
- Likelihood of staff and visitor exposure to infected chicks - Compliance with biosecurity protocols, behavior and training - Likelihood of infection in chicks - Type and material of baskets used to transport infected and Non-infected chicks - Presence and survival of the virus in the environment in the dispatch area	**LOW (medium uncertainty)**	Control measures are normally put in place, but the measures implemented vary between farms

Each of the steps includes a number of risk pathways specified below and in Figure 2.

#### Pathway I. The Likelihood That an Egg Production Site Has Undetected or Unreported, Incubation, or Subclinical HPAI Infections

Egg production sites start operating when breeding stock are moved from over-wintered sites (February) or caught from the wild (until the end of January for England/Wales/Northern Ireland and until the end of February in Scotland). Breeding stock are either kept in an indoor controlled environment (controlled lighting, temperature etc.), which aims to enhance egg production from early March, or a large outdoor enclosure with a net over the top (for wild caught birds). A further three sub-pathways were identified for the Pathway I:

##### 1. Likelihood That HPAIV Is Introduced to an Egg Production Site *via* Breeding Stock Infected/Undetected With HPAI

Released and recaptured pheasants can be infected with AIV by: direct/indirect contact with infected wild birds in their habitat, direct/indirect contact with infected domestic birds in the IP, or coming in contact with a contaminated environment. Pheasants like to live in woodlands, copses and hedgerows. The home range of artificially reared gamebirds depends on the weather and food availability, but they are likely to stay close to releasing sites (10, 12). Since wild waterfowl are the species that most commonly carry AIV, proximity to lakes and ponds increases the risk that pheasants have been in contact with infected wild birds. For AI control purposes, Higher Risk Areas (HRAs) are defined; these are areas with high aggregations or known assemblages of wild water birds (41), as well as other risk factors such as a history of previous outbreaks, positive surveillance results, detections in wild birds, or high density of poultry. Pheasants in HRAs are therefore at increased risk of contact with infected wild birds.

The environment may be contaminated with AIV by feces and respiratory exudates from infected wild birds or *via* decaying carcases of infected dead birds. Outbreaks of HPAI (H5Nx especially derived from Goose/Guangdong lineage) in free-living wild birds are often associated with geographical and chronological proximity to known outbreaks in domestic poultry, which may reflect that transmission can happen from domestic poultry to wild birds as well as from wild birds to domestic poultry (27). Water and bedding materials in the IP may be contaminated with AIV and become a source of infection (28). The habitat of released gamebirds can be contaminated by fomites (e.g., human clothes, footwear, vehicles, and equipment) or *via* contaminated vermin and wild animals from the IP (36, 42), which is often associated with the secondary disease spread (27). Capturing of wild birds is prohibited in disease control/monitoring areas (1, 13). However, released birds that share their habitat with the above areas can be captured from the wild if the site is out of these areas. Cages for captured pheasants can be contaminated with excrements/feathers of infected birds and also be a source of secondary disease spread.

Over-wintered birds can be infected with AIV *via* direct/indirect contact with infected wild birds or fomites (contaminated people and equipment). Commercial game farms and some gamebird keepers keep birds over the winter as breeding stock for the next season. Birds are kept in a large, fenced grass run from June until moving to an egg production site in February. This overlaps with winter migrating season of wild waterfowl species. Grass runs are usually too large to cover with nets, and instead the over-wintered stock are attached with tape or brails (bands attached to the wings) to prevent birds from flying out (*pers comm* with the industry representative). Over-wintering sites located close to the HRA or WBCA (in case of H5N1 in wild birds) have a higher chance that infected wild birds are present. Outdoor grass runs with no netted roof would allow direct contact with infected wild birds and indirect contact through wild bird feces, and the environment, feed, and drinking water can be contaminated with AIV by excrements from infected wild birds.

Incursion *via* fomites depends on the geographical and epidemiological proximity of over-wintering sites to the IP and by the potential for cross-contamination *via* other premises. If an over-wintering site is located in a PZ, there is at least one known infected premises within 3 km, which may be at a varying stage of diagnosis, slaughter, and C&D. If the over-wintering is located in a SZ, the smallest distance at which known infected premises could be located would be 3 km away. In both zones, the likelihood of cross-contamination may be low, as long as there is no movement of people between the premises and no sharing of staff, vehicles, and equipment. However, the likelihood of cross-contamination will increase if the IP is within the same gamebird operator (other rearing sites of the same operator). Multiple rearing sites located nearby may have shared staff, equipment, feed, and vehicles—movements of fomites between the sites can occur, unless rigorous hygiene practices are in place (high uncertainty).

The likelihood that at least one parent bird is infected with HPAI but is not detected before/on arrival at the egg production site depends on the length of infection, viral strain, incubation period, host species, and regular health inspection by farm staff before/on arrival at the egg production site. The onset of disease of HPAI will vary depending on virus strains but has been shown to be 3–4 days post-infection (43). There is medium uncertainty as to whether infected birds show high mortality and clinical signs of disease, since it is highly dependent on circulating strains and host species present; data are limited and outcomes differ depending whether the infection occurred naturally or by an experimental inoculation (see section 3.2 Hazard Identification).

The likelihood of bird-to-bird transmission depends on host species, circulating virus strain and environmental factors such as shared water source and weather conditions (27, 40). The amount and the duration of viral shedding after an AI infection vary depending on host species and virus strains (2). For example, waterfowl infected with H5N2 (HPAI) did not shed virus in feces, despite the detection of the virus from intestine and trachea (35), whilst infection with H9N2 in ducks resulted in viral spread within 2 days post-infection (34). Experimental inoculation of pheasants and partridges with various AIV strains successfully transmitted the virus to birds placed in the same cage *via* a direct and oral-fecal transmission (28). This suggests that it is important to adequately monitor egg production and mortality records and report any changes in health status to veterinarians, especially when an outbreak is confirmed in wild birds or there is a high risk of incursion.

Information on whether regular health inspection before/on arrival at the egg production site is a common practice is scarce. However, any gamebird premises in the PZ will be visited by an official veterinarian and sample collection and testing may be conducted where necessary (44). Visual screening of flock health conditions (e.g., feather condition checks, identification of lethargic, dead birds) may be conducted at the time of catching up from the wild or before transferring over-wintered flocks to the egg production site. Infected birds with clinical signs could be identified at this stage or may not be caught up at all using a trap with feed due to reduced mobility.

We estimated the likelihood of at least one parent bird is infected but not detected and brought into an egg production site as HIGH for infection with mild/no clinical signs and VERY LOW for infection with high mortality and clinical signs with high uncertainty. Theoretically, there should be no gamebirds caught from the wild after February (or March in Scotland), and all the over-wintered stock will be moved to egg production sites before the end of February. Therefore, after these dates, no new parent stock should be introduced, and there should be no risks of AI introduction into egg production sites through infected/undetected breeding stock.

##### 2. Likelihood That HPAIV Is Introduced to an Egg Production Site *via* Infected Wild Birds and Their Fecal Materials

Factors influencing the likelihood that breeding stock in an egg production site is infected *via* direct/indirect contact with infected wild birds include the location of egg production sites, the presence of infected wild birds and the housing system (e.g., outdoor access) of the egg production site. Egg production sites close to HRAs or WBCAs (in case of H5N1 in wild birds) have a higher chance that infected wild birds are present in the area. The likelihood that HPAIV is introduced to a production site *via* infected wild birds is MEDIUM with low uncertainty, if the egg production site has outdoor access (regardless of netting) and (1) is located within two km of an HRA, (2) is within the PZ/WBCA, and/or (3) within an area where positive cases from wild bird surveillance have been reported. The likelihood may decrease toward the end of the winter migration season, which corresponds to the time of the year when the hatchery becomes the busiest. However, there is an ongoing risk of LPAI outbreaks at other times of year, which could mutate to HPAI [e.g., June in 2008 (45), July in 2015 (46) in the UK]. The likelihood for a production site with outdoor access and located out of these zones, or for an indoor production site is estimated as VERY LOW with low uncertainty.

##### 3. Likelihood That HPAIV Is Introduced to an Egg Production Site *via* Fomites From IPs

Factors influencing the likelihood that breeding stock in a production site is infected *via* fomites include the geographical and epidemiological proximity of egg production sites to the IP and the potential for cross-contamination *via* other premises (see 1 above). Indirect contact with AIV *via* human clothing, footwear, vehicles and equipment from the IP is often associated with the secondary disease spread (27). The likelihood will increase if the IP is within the same gamebird operator (other production site or rearing site of the same operator). There is medium uncertainty about whether egg collection pathways and egg transport vehicles are shared within the same operator. Without the application of basic hygiene measures, movement of AIV on staff, equipment, and vehicles between the sites can occur.

Multiple production sites located nearby may have a shared egg collection pathway and may share egg transport vehicles. However, the exact distance at which the hatchery is considered a separate location is not clearly defined. Within the same gamebird operator, it is uncertain whether vehicles are shared or dedicated for bird transportation only. The likelihood is estimated as VERY LOW (medium uncertainty) if the IP is not a commercial gamebird farm, or if the IP is a commercial gamebird farm but is a different operator (no business links). If the IP is part of the same business, the likelihood will increase especially if they share staff, vehicles and equipment (MEDIUM).

#### Pathway II. The Likelihood That a Designated Hatchery Is Contaminated With HPAIV

We assumed that there is HPAI infection present in at least one of the egg production sites. Three further sub-pathways were identified:

##### 4. Likelihood That HPAIV Is Introduced to a Designated Hatchery Through Infected/Contaminated Eggs

###### 4.1. Likelihood That an Infected Bird Lays Eggs.

Experimental inoculation of mallard ducks with H5N1 virus resulted in no clinical signs but a significant reduction in egg production during the first week, which was recovered in the second week (39). H5N1 virus causes high mortality for pheasants and partridge (43), and a drop in egg production is likely (47). Compared to chickens, information on the impact of natural infection, infections with other strains and infections of other gamebird species on egg production is limited. Infected birds may decrease egg production even with LPAI, although this evidence is based on studies in the chicken and the turkey (48–50). The likelihood that an infected bird lays eggs is estimated as HIGH (medium uncertainty) if the circulating AIV causes sub-clinical or no clinical symptoms, and LOW (low uncertainty) if the circulating AIV causes high mortality, clinical signs, and reduced egg production.

###### 4.2. Likelihood That Eggs Laid by Infected Birds are Contaminated With HPAIV but Not Detected.

If infected birds do lay eggs, the eggs may be contaminated with HPAIV before, during or after oviposition (including from other birds), and so they could have internal or surface contamination or both (48, 50–52). Surface contamination is possible *via* the virus excreted in the feces of infected birds and the virus may survive for prolonged periods under certain environmental conditions (51). In experimentally infected birds, some HPAI and LPAIVs can be shed in feces and respiratory secretions as early as 1–2days after inoculation. Some HPAIVs have also been found in meat 1 day after inoculation and in eggs after 3 days (52).

The information on the possibility that HPAIV contaminates eggs laid by gamebird species is scarce (high uncertainty). Studies from the chicken suggested that hens infected with H5 or H7 HPAIV produced virus-positive eggs from 1 or 2 days after infection (50, 53, 54), although not all eggshells were contaminated (50). It is possible that contaminated eggs depart the production site before HPAI would be clinically detected in breeding stock. Therefore, the likelihood of eggs contaminated with HPAIV and not detected is estimated as LOW for gamebirds infected with strains causing high mortality and clinical signs, and the likelihood of eggs contaminated by HPAIV and not detected is estimated as MEDIUM for gamebirds infected with strains causing mild/no clinical signs.

###### 4.3. Likelihood That Virus Survives on the Eggshell or Inside the Egg.

The likelihood is influenced by the viability of HPAIV in the environment. The H5N2 virus was isolated from dead pheasants stored at 4°C for 23 days (35). The survival of viruses on the eggshell depends on the degree of contamination and the effectiveness and reliability of washing and fumigation processes at the production site and/or prior to entry to the hatchery. The exposure to sunlight (e.g., ultraviolet light) could reduce the infectivity of viruses (55).

Eggs collected at the production site are transferred to the hatchery once per week. Grading and sorting eggs according to surface contamination and breakages may be able to mitigate the likelihood that contaminated eggs are delivered to the hatchery. Eggs are normally washed with warm water and sanitized with industry recommended sanitisers before being delivered to the hatchery (pers comm with the industry representative), but there may be variations in the effectiveness of this practice depending on operators. For commercial table eggs, washed eggs may be treated as higher risk because washing can introduce contamination into an egg (56, 57). However, where egg washing is a common practice, washing liquid (disinfectant) should be at a sufficient dilution and an appropriate temperature to destroy the virus and not to draw virus into the shell of egg contents. Fumigation with formaldehyde enables material within the shell to be inactivated but organic material on the shell reduces the efficacy of the fumigation. There is uncertainty about egg washing practices in the gamebird sector. Washing eggs could introduce contaminants into the egg (56), and if they are not treated separately from unwashed eggs, this could raise the likelihood of cross-contamination.

Eggs are unlikely to hatch if infected. There is uncertainty about virus survival time within the egg. The likelihood of virus survival on the eggshell or inside the egg is estimated as LOW (medium uncertainty).

###### 4.4. Likelihood That Infected/Contaminated Eggs are Imported From Outside GB.

It has been reported that 40% of gamebirds released in GB are imported from EU member states (e.g., France, Spain, Denmark) as fertile eggs or as day-old chicks (10, 12). Approximately 70% of red-legged partridges are imported as eggs or as day-old chicks (10). Anyone can purchase gamebird eggs online, which makes it difficult to trace the origin of eggs. It is possible that eggs from infected but undetected birds are imported from EU member states, and these eggs are likely to be washed and fumigated before arrival. HPAI infection without high mortality and clinical symptoms may not be detected without laboratory testing, and it is possible that infected/contaminated but undetected eggs are imported to GB.

It is assumed that eggs entering GB are all inspected at a border. However, there is high degree of uncertainty about how many eggs are purchased online from outside GB and whether they are all traceable. Infected/contaminated eggs may enter GB if the AIV is circulating in the country of origin and if detection of HPAI on source farms is delayed due to the absence of increased mortality or clinical signs of disease. Therefore, the likelihood that infected eggs are imported from outside the UK is estimated as LOW if there is no infection in the country of origin and MEDIUM if there is HPAI infection in the country of origin.

##### 5. Likelihood That HPAIV Is Introduced to a Designated Hatchery *via* Fomites Associated With Egg Movements From the Production Site

###### 5.1. Likelihood of Virus Contamination of/Survival on Trolleys, Reusable, or Disposable Trays.

This will depend on the type and material of egg trays used (plastic or disposable). If eggs from different farms are stored on the same trolleys, it will increase the likelihood of contamination. It will also depend on the frequency and effectiveness of C&D protocol on trays, pallets and trolleys. To our knowledge, there is no published data on the survival of virus on plastic egg trays. The inactivation of influenza A virus depends on the environment (e.g., relative humidity and temperature), type of surfaces, and transmission mode (e.g., aerosol) (58). A human influenza study (59) reported that viable influenza virus (H1N1) was recovered from surfaces for up to one (cotton & microfiber) or two (stainless steel) weeks. However, assuming that the C&D protocol is complied with, and the type/dilution of disinfectant used is sufficient to kill the virus on plastic or metal non-porous surfaces, the likelihood of contamination of and virus survival on trolleys and reusable trays should be VERY LOW (medium uncertainty).

###### 5.2. The Likelihood of Virus Contamination of/Survival on People Transmitting Infection Between Production Sites and the Hatchery.

Personnel at production sites, vehicle driver, hatchery staff facilitating the transfer of hatching eggs into the hatchery, and any staff/visitors who spend time both at production sites and hatchery (including external contractors, visitors, veterinarians) can transmit infection between production sites and the hatchery. Staff and visitor should comply with the company's biosecurity protocols, behaviour, and training. This may include advance warning of arrival and pre-visit questionnaires about bird ownership outside the business, removal of outdoor shoes and showering in and out of the premise. However, due to the seasonal nature of gamebird rearing, casual workers may not be familiar with disease risks and it may be difficult to always evaluate staff background information. Strict biosecurity measures such as the requirement of disposable overalls for vehicle drivers, staff and visitors, allocating a designated car park for staff and visitors, allowing for only one visit per day, and restricting bird ownership of staff could further reduce the likelihood. In the presence of these precautionary steps, the likelihood is estimated to be LOW (medium uncertainty).

###### 5.3. The Likelihood of Virus Contaminating the Outside and Inside of Vehicles Moving Between Production Sites and the Hatchery.

Contamination of vehicles is contingent on virus presence and survival in the environment, which could result in contamination of wheel arches, tire or be transferred to the interior of the vehicle. This contamination could be introduced into the vehicle through contaminated eggs, fomites on staff, or egg trays and trolleys. Small-scale farms may deliver eggs directly from each production site to the hatchery, but large-scale farms may have multiple egg production sites and collect eggs on the way to the hatchery. The egg collection pathway should be explicitly documented, and all eggs from production sites should be identifiable by labeling (trays or trolleys). This would reduce the likelihood of cross-contamination on the collection route.

The exact distance at which the hatchery is considered a separate location is not clearly defined, which may be a challenge for hatchery designation. There is a high degree of uncertainty about the proportion of gamebird operators with multiple production sites on the way to a hatchery, whether they share egg collection pathways, how many vehicles are used for egg collection and whether the vehicles are dedicated for egg transportation only. The likelihood of a contaminated vehicle arriving at a hatchery will increase with increasing numbers of vehicles and the frequency of movements. However, with appropriate C&D of vehicle wheels and arches before entering the curtilage of each farm visited, the likelihood of viral contamination of egg transportation vehicles is estimated as LOW (high uncertainty).

##### 6. Likelihood That HPAIV Is Introduced to a Designated Hatchery *via* Infected Wild Birds or Infected Fecal Material

If HPAI is detected in wild birds, hatcheries located within the WBCA (in case of H5N1 in wild birds) or close to areas where wild waterfowl are found, such as lakes and ponds and/or HRAs, are at higher risk of contamination. This also applies to hatcheries located within the PZ when the IP was infected from wild birds. The environment near or inside the hatchery may be contaminated with HPAIV by excrements from infected wild birds. Therefore, strict biosecurity measures are important to prevent infections from wild birds or infected fecal material. A breach of biosecurity will increase the likelihood of virus introduction into inside the buildings *via* fomites on people or equipment. Hatcheries are operating from mid to late March till mid-June, overlapping with the winter bird migration season.

The likelihood that the HPAI is introduced into the hatchery *via* infected wild birds or infected fecal material is estimated as MEDIUM for a hatchery located within two km of an HRA and/or within a WBCA/PZ, LOW for a hatchery located in an SZ or WBMA (in case of H5N1 in wild birds), and VERY LOW for a hatchery located in an RZ (in case of some H5 strains in poultry) or disease-free zone. The likelihood is also influenced by the level of biosecurity measures in place, which may vary between operators (medium uncertainty). The likelihood may decrease toward the end of the winter migration season, which corresponds to the time of the year when the hatchery becomes the busiest. However, there is an ongoing risk of LP outbreaks at other times of year, which could mutate to HPAI (e.g., June in 2008, July in 2015 in the UK).

#### Pathway III. The Likelihood That HPAIV Is Introduced to Gamebird Rearing Sites

The likelihood that HPAIV is introduced to gamebird rearing sites is contingent on the assumption that there is infection or contamination with HPAI present in the hatchery or on at least one egg, which is transported from egg production sites to the hatchery. If the cross-contamination occurs within the hatchery, we assume that at least one of the day-old chicks is infected or contaminated with HPAI within the hatcher. The likelihood that the HPAIV is introduced to the gamebird rearing site through the movement of day-old chicks from the designated hatchery involves the following three sub-pathways:

##### 7. Likelihood of Cross-Contamination Within a Designated Hatchery

###### 7.1. Likelihood that the HPAIV Is Transferred to Other Eggs, Trays and Trolleys During Egg Sorting and Grading, Resulting in Cross-Contamination of Eggs.

The likelihood that the virus is transferred to equipment depends on the viral load on the surface of the eggshell and whether or not eggs are dirty, transfer of eggs in trays into trolleys is done mechanically or manually, eggs are touched or egg breakages occur. If multiple trays from different farms are stored together on trolleys or pallets, this will increase the likelihood (this is probably a common practice). Eggs are typically graded and washed at the production site prior to arrival at the hatchery which reduces surface viral load. However, washing eggs may introduce contaminants into the egg (if not dried immediately) and make detection of such contamination more difficult (56).

Virus survival on eggshell depends on the degree of contamination and the effectiveness and reliability of processes introduced to remove this contamination (via washing or fumigation) at the production sites and/or prior to entry to the hatchery. Epidemiological findings from the past HPAI outbreaks suggest that the virus could survive on plastic egg trays for hours, and heavy fecal load will extend this period. The virus can be transferred to clothes and hands of staff on-site, and cross-contamination to eggs and equipment (trays and trolleys) can occur if handled with contaminated hands. The ability to adequately disinfect trays/trolleys depend on the type and material of egg trays used (plastic or disposable) and the frequency and effectiveness of C&D protocol on trays, pallets, and trolleys.

Scientific studies on the survival of HPAIV on plastic egg trays are scarce, which resulted in a high degree of uncertainty around this likelihood. Studies on human influenza virus [H1N1: (59)] and influenza A virus in general (58) reported that viable influenza virus was recovered from various surfaces (e.g., cotton & microfiber, stainless steel: also see section 5.1). If the HPAI infection is confirmed on at least one of the source farms, eggs from the IP and eggs that have been mixed with these eggs at any points in the hatchery may be considered contaminated. We assumed that C&D protocol is complied with and that the type/dilution of disinfectant utilized is sufficient to kill the virus on plastic or metal non-porous surfaces. With appropriate C&D, the overall likelihood is estimated as LOW (high uncertainty).

###### 7.2. Likelihood that Cross-Contamination of Eggs Occurs Within the Egg Storage and in the Incubator.

Eggs are stored and incubated on a tray, which should create some space between eggs (not touching neighboring eggs). Therefore, one plausible scenario for the cross-contamination of eggs could occur during these periods is probably when eggs break on a tray and the broken egg shells and contents drop down on to the eggs below. The contents of eggs from infected birds may be contaminated with the virus (48). It is uncertain how often egg breakage would occur at the egg storage, but infected eggs can have poor quality egg surface (60), which can contribute to the egg breakage. Egg candling for fertility (after 7–8 days of incubation) may act as a potential source of cross-contamination if the equipment (light) is contaminated with HPAIV (e.g., the virus is transferred from the surface of a contaminated egg to uncontaminated eggs).

The likelihood estimated for cross-contamination of eggs within the storage and the incubator is LOW (low uncertainty). Appropriate room temperature and conditions and protocols to prevent and manage egg breakages will reduce the likelihood. If feasible, reducing the proximity between trolleys or pallets with eggs from different source farms and fumigation/room fogging with government-approved disinfectant will also reduce this likelihood. The likelihood of cross-contamination through egg candling will decrease if the equipment is cleansed and disinfected regularly.

###### 7.3. Likelihood of Cross-Contamination of Eggs or Infection of Chicks Within the Hatcher.

This depends on virus survival time on the surface of eggs and the presence of at least one infected/contaminated egg in the hatcher. Assuming that the appropriate control measures such as adequate C&D and no egg breakage are in place, this is already a very low likelihood. Eggs in the hatcher are placed on a big plastic basket using a sheet to prevent an egg breakage. Virus surviving on the egg surface may contaminate handlers, sheet or a basket, resulting in cross-contamination of eggs. The mechanical transfer of eggs from trays into baskets will reduce opportunities for further contamination of eggs.

Studies on chicken have shown that infection with AI decreases the hatchability of eggs (52), but another study in chicken reported that a small number of HPAI (H7) infected embryos remained viable for 18 days after experimental inoculation (50). There is limited information on the egg hatchability and post-hatching infection in gamebirds (medium uncertainty). Experimental inoculation of HPAI H5N1 in Mallards significantly decreased egg production but did not affect egg weight, fertility, and early death (39). Chick growth rate was also not affected in the same study, although this study did not report whether chicks were infected. High mortality is expected if chicks are exposed to HPAIV and infected after hatching. However, eggs from infected parents may successfully hatch and chicks could grow normally, if there was no embryo or post-hatching infection.

Maintaining appropriate conditions within the shared airspace (which include appropriate ventilation and temperature of the room) reduces opportunities for virus survival and the subsequent infection of hatching chicks. However, very high airspeeds and production of chick fluff increase the likelihood of cross-contamination within the hatcher. If fumigation is done just before hatching, this could reduce the viral load. The likelihood is estimated as LOW (medium uncertainty).

##### 8. Likelihood That HPAIV Is Introduced Into Gamebird Rearing Sites *via* the Movement of at Least One Infected/Contaminated Day-Old Chick

It is unlikely that chicks are exposed to high infectious dose at this stage, but exposure to highly virulent strain should cause clinical symptoms or a sudden death within 24 h after hatching. There is medium uncertainty in the likelihood of post-hatching infection of chicks from surface-contaminated eggs. Studies in chicken reported that hatched chicks from infected premises may not necessarily be infected with the virus (52). At this stage, all eggs from different production sites may be mixed and impossible to trace back to the source farm. If one of the source farms is confirmed with HPAI infection, it may be necessary to assume that all the eggs/chicks in the hatchery are contaminated with HPAI (though this depends on the stage of the outbreak). The likelihood is estimated to be LOW (medium uncertainty).

##### 9. Likelihood of HPAI Transmission *via* Fomites Associated With the Movement of Day-Old Chicks

Fomites include (i) contaminated equipment which is used to deliver day-old chicks to rearing sites (e.g., trays and baskets), (ii) contaminated people (e.g., staff, external contractors, visitors, veterinarians, pharmaceutical representatives, vehicle drivers) and (iii) contaminated vehicles. The likelihood of contamination of equipment and people with the virus depends on the presence of infection in chicks, the chance of equipment, people, and vehicles being exposed to infected chicks (already low likelihood), the compliance with biosecurity protocols, the behavior and training of staff and visitors, the type and material of trays and baskets used to transport infected and non-infected chicks. If large numbers of chicks from different farms are transported within the same basket or held close to each other, it will increase the likelihood of contamination.

The likelihood of contamination of the interior or exterior of vehicles also is contingent on the presence and survival of the virus in the environment (e.g., from fecal material or fomites transferred by vehicles or staff) as well as the compliance with an appropriate C&D protocol. This is particularly true in the dispatch area of the hatchery, which could result in contamination of wheel arches, tire or be transferred to the interior of the vehicle. Virus survival in the environment is also a key driver (see previous sections 5.1 and 7.1). However, with the compliance with biosecurity protocols and appropriate C&D at the entrance of rearing sites, the likelihood of HPAIV introduction into gamebird rearing sites *via* fomites is estimated as LOW (medium uncertainty).

### Overall Likelihood Estimates

The overall likelihood of introduction of HPAI into a designated hatchery that is located in a PZ, SZ, RZ (in case of some H5 in poultry), or WBCA (in case of H5N1 in wild birds) and receives eggs from production sites located in a disease-free zone is estimated as LOW (high uncertainty). The likelihood of introduction of HPAI into a designated hatchery *via* hatching eggs from production sites located in a PZ, SZ, RZ, or WBCA is also estimated as LOW. However, the likelihood will increase if the gamebird capturing area is near the HRA, WBCA, and/or near IPs (in the case of pheasants). Assuming that HPAI has been detected in poultry or gamebird farms, and/or that HPAI is present in the wild bird population, the likelihood of introduction of HPAI infection into a gamebird rearing premises *via* movements of live day-old chicks from a designated gamebird hatchery is LOW (medium uncertainty).

### Consequence Assessment

If an undetected incursion of HPAI into a hatchery were to occur, there would be a risk of cross-contamination of eggs and subsequently day-old chicks. In the event of an undetected introduction of HPAI into a gamebird rearing site, there would be a risk of cross-contamination of chicks and poults on the site. These events would result in significant economic loss and animal welfare issues as well as the potential for onward transmission of the outbreak within GB. This is beyond the scope of the current work, hence it was not explored in detail.

## Discussion

The outbreaks of HPAI on GB gamebird farms in 2017 and 2021 had serious impacts in the gamebird industry. The outbreaks in 2017 involved multiple rearing premises associated with a single operator, resulting in significant economic impacts. The primary incursions into the gamebird premise for both 2017 and 2021 outbreaks are considered to have originated from wild birds (3, 8), but the subsequent spread that occurred after the outbreak in 2017 was associated with business activities (8). It also revealed that the structure of the gamebird industry was not well-understood by the government or other stakeholders (7), making it challenging to conduct rapid veterinary risk assessments as part of disease control efforts during an outbreak emergency.

In this study, VRAs were used to estimate the likelihood of HPAI incursion into the gamebird sector *via* designated hatcheries, through the movement of hatching eggs and day-old chicks. The work was commissioned by Scottish Government Animal Health and Welfare Division after the 2016/17 outbreak, with the aim to use the VRAs to underpin designation criteria for the gamebird hatchery. As part of this process, the information about the general structure of the gamebird industry in GB was obtained in order to identify risk pathways (as shown in Figure 2). We estimated the overall likelihood of the HPAIV introduction into a designated hatchery through hatching egg movements to be LOW (rare but could occur) with high uncertainty. The likelihood of onward transmission of the HPAIV into gamebird rearing sites from a designated hatchery through day-old chick movement is also considered to be LOW with medium uncertainty.

HPAIV can be introduced to the gamebird hatchery through contaminated hatching eggs from the production site, fomites associated with egg movements between contaminated egg production sites and designated hatcheries, and indirect/direct incursion from infected wild birds. The likelihood levels of these pathways depend principally on the level of biosecurity and the proximity of the site to known areas of wild bird infection or IP. Stringent biosecurity measures and regular health inspection of breeding stock are essential to prevent the introduction of HPAIV, but this may pose a challenge for hatcheries that receive eggs from multiple satellite farms with different locations and different operators. The likelihood levels also depend on the season. In recent HPAI outbreaks in the UK, direct/indirect contacts with infected wild birds has been considered as the most likely route of virus introduction into domestic poultry (3, 8). Therefore, the likelihood of HPAI of incursions into the egg production site and the designated hatchery *via* infected wild birds (sub-pathway 2 and sub-pathway 6) is estimated to decrease toward the end of winter migrating season. Catching of breeding stock from the wild and the movement of over-wintered stock to the egg production site has normally been completed when gamebird hatcheries start operating in mid-March or April. This means that the virus introduction to the egg production site *via* infected/undetected breeding stock (sub-pathway 1) and subsequent introduction to the hatchery (sub-pathway 4 and 5) is only possible when there are undetected LPAI infections in breeding stock that mutate to HPAI.

The current criteria for hatchery designation were developed based on commercial poultry farms, which may not apply to the gamebird sector due to differences in hatchery operation systems and bird species kept. One of the most striking differences in management practices between the gamebird and commercial poultry sectors is that gamebird breeding stock are either caught from the wild (pheasants only) or kept over-wintered on an outdoor grass run. This would increase the chance that gamebirds have direct or indirect contact with wild birds and their excrements, making the implementation of appropriate biosecurity measures challenging. The premises at greatest risk of interaction with wild birds are those located near HRA (41) or the PZ (if the IP has arisen from wild bird infection). Released gamebirds and wild waterfowls are likely to share their habitat, and scattered feed for released birds may attract wild birds, especially when there is food shortage in the wild. Spilled feed for over-wintering stock and ponds on duck farms may also attract wild birds (3).

Some gamebird hatcheries may be located in the same location as or close proximity to the egg production or the rearing sites. Hatchery designation may not be required if these sites are considered as the same premises, although the distance at which the two premises are considered as a separate place is not clearly defined in the designation criteria. These operators may also receive eggs from satellite farms that are in separate locations, and hatchery designation is necessary in this case. This would become a problem for an operator if staff, vehicles and equipment are regularly shared between the hatchery and other production sites located in the same place. The information on how gamebird farms in GB operate in terms of staff allocation and staff sharing is scarce, and control measures put in place may vary significantly between large-scale modern gamebird farms and small-scale or gamebird farms using traditional production and husbandry approaches.

Additionally, there are no published data or documents for biosecurity measures implemented inside the hatchery (e.g., measures implemented at egg arrival, egg storage, incubator, hatcher, and package/dispatch area). Industry representatives suggested that egg washing and sanitizing was a common practice in the gamebird industry, but information about the effectiveness of this practice is scarce. This is probably another area where large variations exist between operators. In general, biosecurity and hygiene levels in the gamebird sector are considered sub-optimal compared to the commercial poultry sector (10), due to their differences in operation systems, such as outdoor access for breeding stock and catching adult birds from the wild. Indeed, the HPAI outbreak in a gamebird farm in 2021 was partly due to poor biosecurity measures (3). A report on visits to five different UK gamebird premises also described biosecurity measures in UK game farms as either non-existent or very limited, highlighting issues such as a poor biosecurity protocol for staff/visitors and the lack of preventative measures against wild birds in the outdoor run (20). Further studies or surveys on typical management practices and biosecurity measures in the gamebird hatchery, as well as studies to investigate the effectiveness of egg washing practice would reduce uncertainty levels for Pathway II and III and allow for more accurate estimates for the likelihood levels.

Due to the seasonal nature of gamebird rearing, casual seasonal workers who are not familiar with disease risks may not always comply with biosecurity protocols. It is uncertain whether each farm has specific employee requirements such as the requirement for no bird ownership at home or no interaction with birds outside the business. It has been reported that backyard flocks that have links to commercial poultry farms [e.g., close location (61), shared personnel (62)] are known to increase the risk of disease spread between the sectors. Staff allocated to work at multiple locations may increase the risk of AI transmission, as their clothes and footwear can be contaminated with the virus by human-bird interactions (63, 64).

There are situations in which gamebird farms can be at lower risk of disease spread compared to the commercial poultry sector. An analysis of upland gamebird farm management practices in the United States indicated that upland gamebird farm normally had a single independent owner having all the production sites on one premise, being geographically distant to neighboring farms, and having a dedicated staff for each site (42). Those farms usually did not share contract veterinarians with other gamebird farms. The same study also found that individual employees of these gamebird farms performed almost all farm tasks such as capturing of breeding stock, C&D and land management, and vehicles and equipment were not usually shared with other farms (42). This would reduce the risk of disease spread to other gamebird farms. However, it is uncertain how much of these risk reduction strategies are applied on GB gamebird farms.

Recent outbreaks in the UK have shown that the infection with H5N1 and H5N8 virus strains caused high mortality to gamebird species as well as other poultry species (3, 8). However, there were cases of HPAIV infection in gamebirds that did not involve high mortality and severe clinical symptoms (35, 61). Visual health inspections may be in place at the egg production site, but routine laboratory testing for AIV is unlikely to occur in the gamebird sector. Ssematimba et al. (62) suggested a mortality-based model for a more targeted surveillance strategy, which triggers an alarm to initiate diagnostic interventions based on increased flock mortality rates (e.g., H5N1). Indeed, active AI surveillance programmes implemented in the United States in the early 2000s led to the detection of the majority of AI outbreaks in commercial gamebird farms and have been proven useful in collecting information on certain management practices (29). An early detection of AI is a key to minimize the impact, but if there are HPAI outbreaks that do not involve high mortality and severe clinical symptoms in gamebirds (35, 61), the detection of infection may only be possible by routine testing for AI.

The estimated likelihood levels were based on the assumption that there is full compliance with preventive biosecurity measures for AI. These measures include no shared vehicles, staff, equipment and pathways between the gamebird and commercial poultry sectors, or between different gamebird operators, effective C&D, and a well-documented protocol for staff and visitor biosecurity (21). Additionally, adequate record-keeping/traceability, separation of restricted production, and special marking of production are required (1, 13). We used this assumption because enhanced biosecurity is a pre-requisite for hatchery designation; i.e., gamebird hatcheries are only allowed to continue their operation as a designated hatchery during HPAI outbreaks as long as enhanced biosecurity measures and additional requirements are in place. Any breach of the government's protocol would result in the increase in the risk of disease incursion and subsequent transmission in the sector. However, observations made in recent outbreaks suggest that biosecurity is not always implemented appropriately in the gamebird sector (3), and there may be variations between operators. Gamebird hatcheries that do not meet the requirement of designation cannot continue operating during HPAI outbreaks, which affects their associated operation sites such as egg production sites and rearing sites. Further studies are warranted to improve our understanding of the typical biosecurity measures implemented in the gamebird sector, behavioral drivers for biosecurity implementation and variations of these measures between operators. Such studies would help identify farms at a higher risk of disease introduction and transmission and provide suggestions for the improvement of management practices in order for hatcheries to be safely designated in the future.

## Conclusion

Several differences between commercial poultry and the gamebird sector highlight why effective biosecurity and disease control measures in this sector can be challenging. Any uncertainties identified in this study were due to the paucity of data regarding gamebird species, and the data from chickens are not always applicable. There was a high degree of uncertainty about the likelihood of HPAI infection, management practices of gamebird farms in GB, and biosecurity measures put in place. The likelihood estimates are based on the assumption that appropriate control measures are put in place, but the level of implementation may vary with factors such as farm size and management practices. This means that the likelihood estimates presented in this paper are anticipated to be the best-case scenario. Not all hatcheries in GB can meet the requirement for hatchery designation, which could have a significant impact on the industry. However, the limited availability of the data on biosecurity practices of the gamebird sector made it difficult to identify farms at a higher risk.

There are also specific aspects to be considered to reduce the risk. For hatchery designation, it is important to identify the type of hatcheries (e.g., whether a large-scale recognized gamebird hatcheries or gamebird keepers' hatchery in their estate), how they source eggs (either from importation, their over-wintered stock or wild-caught breeding stock) and where they capture breeding stock. Mitigation strategies should include no bird catching during an AI outbreak, monitoring of breeding stock health condition, biosecurity measures to prevent direct/indirect contact with wild birds, documentation of egg origin, and adequate C&D of clothes/boots, equipment, and vehicles.

## Data Availability Statement

The original contributions presented in the study are included in the article/Supplementary Material, further inquiries can be directed to the corresponding author/s.

## Author Contributions

MF, HA, LB, and IB conceived and designed the study. MF performed the analysis. HA, LB, and IB reviewed the VRAs. MF wrote the manuscript. All authors contributed to manuscript revision, read, and approved the submitted version.

## Funding

This work was funded by the Scottish Government Rural and Environment Science and Analytical Services Division (RESAS), as part of the Center of Expertise on Animal Disease Outbreaks (EPIC).

## Conflict of Interest

IB was employed by Animal and Plant Health Agency. The remaining authors declare that the research was conducted in the absence of any commercial or financial relationships that could be construed as a potential conflict of interest.

## Publisher's Note

All claims expressed in this article are solely those of the authors and do not necessarily represent those of their affiliated organizations, or those of the publisher, the editors and the reviewers. Any product that may be evaluated in this article, or claim that may be made by its manufacturer, is not guaranteed or endorsed by the publisher.
