# Supplementary material for: Assessing the Likelihood of High Pathogenicity Avian Influenza Incursion Into the Gamebird Sector in Great Britain via Designated Hatcheries
Source: Front Vet Sci. 2022 Apr 22;9:877197. doi: 10.3389/fvets.2022.877197 (PMC9072826; doi:10.3389/fvets.2022.877197)
Supplement: Supplementary file 1 [file Data_Sheet_1.pdf]

Questions to the gamebird farm manager

What percentages of gamebird eggs imported from other member states? (According to Defra's document in 2006, 50% are imported).
When are the egg importations most likely occur?
Do gamebird hatcheries usually accept eggs from different source farms?<br>Or mainly from their own laying sites?
Are eggs from different sources separated? (Is each egg traceable to its source farm?)
What is the typical structure of the gamebird farm? In other words, how many different sites (pens) do they usually have within the same operators?<br>e.g. laying site, hatching site (hatchery), (brooding site?), rearing site, releasing site..
Are a hatchery and rearing sites usually in separate locations?
What is a typical housing system for these sites (outside only, inside with access to outside, inside only)?
Is an outside enclosure usually netted?
When are birds usually released in the wild?
When are birds usually captured?
How are the birds captured from the wild?
What percentages of gamebird farms in GB recapture released birds, and what percentages of farms keep birds overwintered?
Are there many farms do both overwintering and recapturing?
What is a typical enclosure for the birds over-wintered for breeding (outdoor enclosure or indoor cages)?
What is a typical enclosure for the birds for breeding (outdoor enclosure or indoor cages)?
Are eggs collected from the laying site every day? If not, how often?
Are there any farms keeping eggs with hens instead of using hatcheries?
